# Microarrays for Public Health: Genomic Epidemiology of Tuberculosis

**DOI:** 10.1002/cfg.191

**Published:** 2002-08

**Authors:** Jamila Shafi, Peter W. Andrew, Michael R. Barer

**Affiliations:** Department of Microbiology and Immunology Medical Sciences Building PO Box 138 University Road Leicester LE1 9HN UK

## Abstract

In response to a large local school-based outbreak of tuberculosis, we have been evaluating the utility of microarray bacterial genomic analysis in outbreak management. After initial comparison of the isolate from the index case with *Mycobacterium tuberculosis*  H37Rv, it was possible to design robust PCRs directed towards strain-specific deletions. Rapid PCR analysis of isolates proved valuable in determining whether or not other isolates were compatible with the outbreak strain and further microarray studies revealed genetic markers that could be used to discriminate between locally circulating strains.We suggest that this approach forms the basis for developing rapid local genotyping schemes applicable to *M. tuberculosis* and that application to other pathogens warrants consideration.


**Comparative and Functional Genomics**
*Comp Funct Genom* 2002; **3**: 362–365.
Published online in Wiley InterScience (www.interscience.wiley.com). **DOI:** 10.1002/cfg.191

**Conference Review**

### Microarrays for public health: genomic epidemiology of tuberculosis

Jamila Shafi, Peter W. Andrew and Michael R. Barer*
*Department of Microbiology and Immunology, Medical Sciences Building, PO Box 138, University Road, Leicester LE1 9HN, UK*

**Correspondence to:*
*Michael R. Barer, Department of Microbiology and Immunology, Medical Sciences Building, PO Box 138, University Road, Leicester LE1 9HN, UK.*
*E-mail: mrb19@le.ac.uk*

Received: 31 May 2002
Accepted: 12 June 2002

### Abstract

**In response to a large local school-based outbreak of tuberculosis, we have been evaluating the utility of microarray bacterial genomic analysis in outbreak management. After initial comparison of the isolate from the index case with *Mycobacterium tuberculosis* H37Rv, it was possible to design robust PCRs directed towards strain-specific deletions. Rapid PCR analysis of isolates proved valuable in determining whether or not other isolates were compatible with the outbreak strain and further microarray studies revealed genetic markers that could be used to discriminate between locally circulating strains. We suggest that this approach forms the basis for developing rapid local genotyping schemes applicable to *M. tuberculosis* and that application to other pathogens warrants consideration. Copyright © 2002 John Wiley & Sons, Ltd.**

**Keywords:** bacterial infection; epidemiology; infection control

We have been exploring the degree to which microarray analyses can be applied to investigate outbreak strains of *Mycobacterium tuberculosis*. The work was provoked by a large local school-based outbreak of tuberculosis and we identified the following objectives in asking what microarray analyses might have to offer in this context:

1. To determine whether the outbreak strain carries genetic markers that could be used for its rapid and specific detection in clinical samples.
2. To study the genome of the outbreak strain for distinctive features that might explain the clinical and epidemiological features of the outbreak.

Our initial results gave a strong indication that microarray genomic analysis can provide the basis for rapid recognition of tuberculosis cases as outbreak or non-outbreak cases by strain-specific PCR, and that a coherent view of the genealogy of locally circulating strains may be obtained. This information has proved of considerable interest to the local public health control effort, principally because results can be obtained on a time-scale compatible with the disease control decision-making process; contrasting with the retrospective analysis produced by most conventional strain-typing studies. Our preliminary work indicates that the approach may be applicable to the control effort of any local tuberculosis problem and that it might also be applicable to rapid resolution of public health issues relating to other infections. Here we review the background to, and key features of, this approach and discuss its potential further applications.

Microarrays have been used extensively to study relationships between bacterial strains and can provide a coherent view of relatedness and phylogeny. One study on a set of *M. tuberculosis* isolates for which epidemiological data were available included some microarray work but only three related isolates were studied (5). As far we are aware, the utility of this approach for outbreak analysis and management has not been evaluated previously.

The approach we have established is illustrated in Figure 1. The initial rate-limiting step is obtaining sufficient genomic DNA for the first round array hybridization. In the ORF amplicon-based array we have used, this reveals potential gene deletions (and duplications). These are then confirmed

Copyright © 2002 John Wiley & Sons, Ltd.

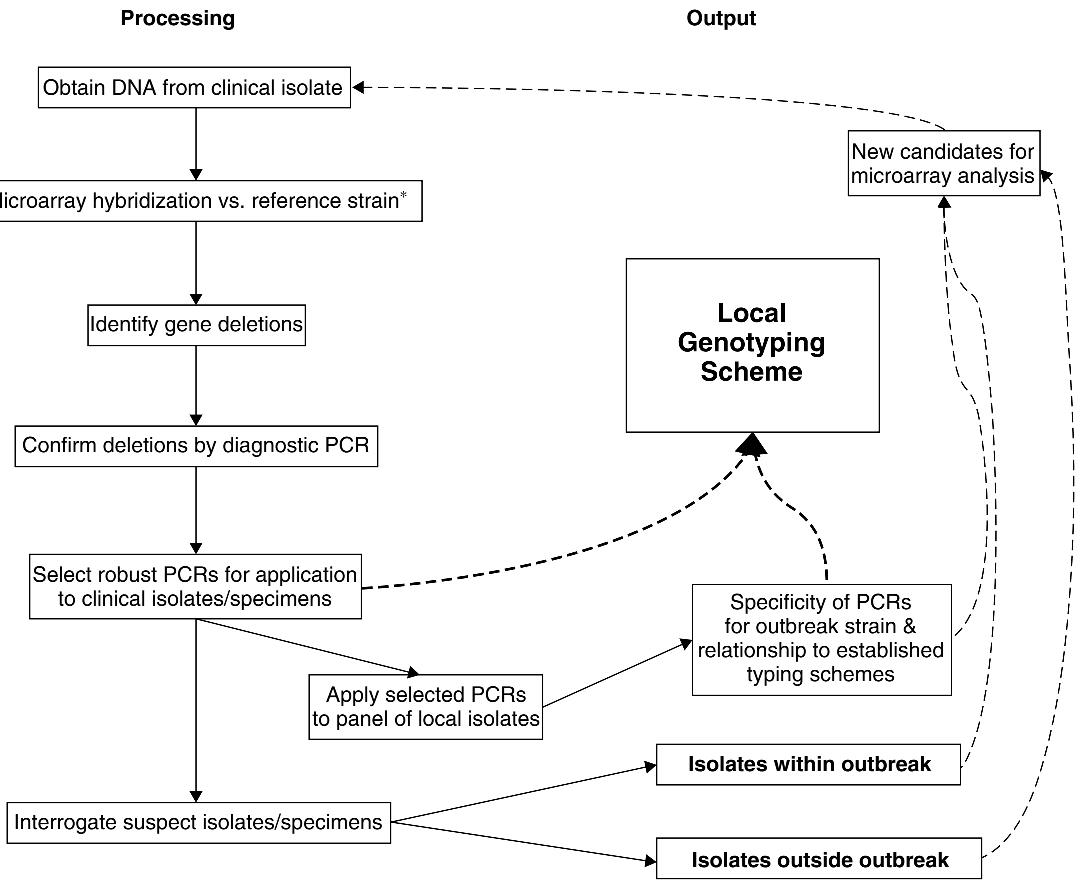

**Figure 1.** Microarray-derived genotyping for public health. An iterative process is outlined in which array hybridizations are used to screen for strain-specific genetic markers. Dashed lines indicate stages occurring after the first round array analysis. As more markers are identified, so a more robust locally applicable genotyping scheme is obtained. *H37Rv in our case, but ideally this should reflect the known gene pool for the *M. tuberculosis* complex by diagnostic PCR. Once this has been achieved, specific deletion-targeted PCRs can be designed and evaluated. Since the primers for such analyses are already available for the manufacture of the arrays, this evaluation is very rapid and the resultant selected PCRs can be applied directly to new isolates or to acid-fast bacilli-positive specimens. Such analyses might also be possible on smear-negative samples, although the public health issues are lessened here because of the weaker association with transmission in such patients. Even when only one or two deletion-directed PCRs have been evaluated, these can be useful, since the detection in a test isolate of a gene that has been deleted in the outbreak strain makes it unlikely (but not impossible) that the former came from the outbreak. In our outbreak strain, from an initial 11 possible deletion loci compared to H37Rv, five robust diagnostic PCRs were established. One borderline weak array signal proved not to be a deletion and three of the deletions involved two adjacent genes. It should be noted that PCR results can be interpreted with confidence, as analyses yield small or large amplicons depending, respectively, on the presence or absence of the target deletion.

In order to determine the discriminatory power of detecting strain-specific deletions by PCR, we applied this analytical approach to local isolates from a period up to 2 years prior to the outbreak. These represented all the main IS6110 RFLP types isolated and were tested blind, together with other isolates from the outbreak. The five PCRs unequivocally detected all of the outbreak isolates (identified as such by both epidemiological

Copyright © 2002 John Wiley & Sons, Ltd.

and RFLP data). Two strains with RFLP patterns indistinguishable from the outbreak strain, and considered to reflect presence of the strain in the community prior to the outbreak, also tested positive for all five deletions. Of the other strains tested (∼50), isolates with three or less deletions in common with the outbreak strain showed no epidemiological connection with the outbreak. A large group with three outbreak strain deletions in common with each other included diverse RFLP types.

We also applied PCR analysis to new local isolates where our public health colleagues wanted to know whether the case was connected to the outbreak. In each of the seven isolates tested so far, absence of two or more outbreak deletions has allowed us to unequivocally exclude this possibility.

Both applications of the deletion-directed PCRs allowed us to recognize isolates that we felt were appropriate for further microarray analyses. The results of these analyses were useful, firstly in identifying further genetic markers of locally circulating strains and secondly in allowing us to recognize possible genealogies of the isolates. When matched with the epidemiological and clinical data, it was possible in several cases to construct coherent sequences for the evolution of the strains observed.

While a more detailed analysis of these results is in progress, our initial impression is that this iterative analytical approach rapidly yields information that can inform the public health control process. Further, with the identification of additional marker genes for locally circulating strains, a more and more discriminatory panel of diagnostic PCRs becomes available for rapid local isolate/specimen analysis. A major part of our further analysis will include a detailed review of the relationships between our deletion analyses and established typing scheme results.

As far as we are aware, microarrays have not previously been used to facilitate bacterial strain recognition during the course of an outbreak. Recent bacterial genomics studies have mainly concentrated on the evolution of currently circulating stains of the *M. tuberculosis* complex [5], *Staphylococcus aureus* [3], *Streptococcus pyogenes* [6], *Streptococcus pneumoniae* [4], *Escherichia coli* O157 [1] and *Campylobacter jejuni* [2]. Apart from PCR studies to validate the microarray results, these studies have used full genomic analyses throughout. While this presents little problem for rapidly growing bacteria, requirement for substantial quantities of genomic DNA places major time and logistic constraints on the analysis of *M. tuberculosis* isolates; the PCR analyses we have established effectively circumvent this problem.

The utility of the initial microarray analysis of an outbreak strain is of course, limited by the degree to which the array reflects the gene pool of the target organism. While it is generally agreed that lateral gene transfer is not a significant phenomenon in the *M. tuberculosis* complex (hence incoming 'novel' genes should not present a problem), sequencing has demonstrated that CDC1551 has at least 20 more ORFs than H37Rv and these were not represented in our analysis. Ideally, we would like to represent the *M. tuberculosis* gene pool completely in future analyses, but the extent of this (including the possible presence of 'extra' genes in our outbreak strain) has yet to be determined.

The ORF amplicon microarray we used here offers two major advantages: (a) it is a relatively low-cost system; and (b) initial primer sets for downstream analysis are immediately available. In comparison to oligonucleotide arrays, this approach has lower resolving power (i.e. the deletions have to involve a substantial proportion of the amplicon) and presents fewer opportunities for internal result validation.

Analysis of our results in relation to the biological properties of the outbreak strain is at a very early stage. None of the deleted ORFs have provided a clear clue to the strain's pathogenicity. Interestingly, none of the possible progenitor strains detected amongst community isolates seems to have spread extensively, so it is attractive to speculate that genomic alterations between these and the outbreak strain may have been critical to its epidemic potential. Global analyses of gene deletions in larger strain sets of *M. tuberculosis* and other pathogens will also allow us to define a minimum gene set compatible with virulence. In the future, microarray analysis of gene expression in the outbreak strain may provide a further level at which features of the outbreak strain might be related to its public health significance.

At present there are two key questions relating to the approach we have established; does the approach offer an economic and practical alternative or complement to other epidemiological isolate analytical tools, and can the approach be usefully applied to other pathogens?

Copyright © 2002 John Wiley & Sons, Ltd. 

### Acknowledgements

This work was supported by a grant from MediSearch (Leicester). The microarray analyses were performed at the BμG@S (the Bacterial Microarray Group at St George's) under the ever-supportive supervision of Philip Butcher, Jason Hinds and Richard Stabler, and we thank The Wellcome Trust for funding their work. We gratefully acknowledge many helpful discussions with our colleagues in Public Health Medicine, especially Philip Monk, Gerry Bryant and Debbie Modha. We thank Peter Gale, Hemu Patel and Geoff Brooks and their colleagues for providing us with the isolates, and all the members of the Leicester tuberculosis outbreak team, without whose contributions this work would not have been possible.

### References

1. Call DR, Brockman FJ, Chandler DP. 2001. Detecting and genotyping *Escherichia coli* O157:H7 using multiplexed PCR and nucleic acid microarrays. *Int J Food Microbiol* **67**(1–2): 71–80.
2. Dorrell N, Mangan JA, Laing KG, *et al*. 2001. Whole genome comparison of *Campylobacter jejuni* human isolates using a low-cost microarray reveals extensive genetic diversity. *Genome Res* **11**(10): 1706–1715.
3. Fitzgerald JR, Sturdevant DE, Mackie SM, Gill SR, Musser JM. 2001. Evolutionary genomics of *Staphylococcus aureus*: insights into the origin of methicillin-resistant strains and the toxic shock syndrome epidemic. *Proc Natl Acad Sci USA* **98**(15): 8821–8826.
4. Hakenbeck R, Balmelle N, Weber B, Gardes C, Keck W, de Saizieu A. 2001. Mosaic genes and mosaic chromosomes: intra- and interspecies genomic variation of *Streptococcus pneumoniae*. *Infect Immun* **69**(4): 2477–2486.
5. Kato-Maeda M, Rhee JT, Gingeras TR, *et al*. 2001. Comparing genomes within the species *Mycobacterium tuberculosis*. *Genome Res* **11**(4): 547–554.
6. Smoot JC, Barbian KD, Van Gompel JJ, *et al*. 2002. Genome sequence and comparative microarray analysis of serotype M18 group A *Streptococcus* strains associated with acute rheumatic fever outbreaks. *Proc Natl Acad Sci USA* **99**(7): 4668–4673.

Copyright © 2002 John Wiley & Sons, Ltd.
